# Features and Legal Practice of Dried Blood Spot Card Biobanking in Europe: Balancing Research Potential with Parental and Children’s Rights

**DOI:** 10.3390/ijns12030064

**Published:** 2026-08-05

**Authors:** Sophie ter Braak, Mette Nyegaard, Christian Munch Hagen, Marie Bækvad-Hansen, Madara Auzenbaha, François Boemer, James R. Bonham, Patricia Borde, Ian Brincat, David Cheillan, Vera Frankova, Leifur Franzson, Ksenija Fumić, Urh Groselj, Tom L. G. M. van den Kerkhof, Riikka Kurkijärvi, Giancarlo la Marca, Tatjana Milenković, Olve Moldestad, Vyacheslav Mitkin, Florentina Moldovanu, Marios Vogazianos, Jürgen G. Okun, Lene Sörensen, Gulnara Svyatova, Ildikó Szatmári, Maja Raičević, Karit Reinson, Trine Tangeraas, Alma Toromanović, Laura Vilarinho, Svetlana Vorslova, Raquel Yahyaoui, Maximillian Zeyda, Dianne Webster, Peter C. J. I. Schielen

**Affiliations:** 1 International Society for Neonatal Screening, Reigerskamp 273, 3607 HP Maarssen, The Netherlands; ampsophieterbraak@gmail.com; 2 Department of Congenital Disorders, Statens Serum Institut, Artillerivej 5, DK-2300 Copenhagen, Denmark; meny@ssi.dk (M.N.); unc@ssi.dk (C.M.H.); mabh@ssi.dk (M.B.-H.); 3 Children’s Clinical University Hospital, Riga Stradiņš University, LV-1007 Riga, Latvia; madara.auzenbaha@rsu.lv (M.A.);; 4 CHU Liege, 4000 Liège, Belgium; f.boemer@chuliege.be; 5 Sheffield Children’s Hospital, Sheffield S10 2TH, UK; j.bonham@nhs.net; 6 Laboratoire National de Santé, 3555 Dudelange, Luxembourg; 7 Mater Dei Hospital, TAL-QROQQ Msida, Malta; 8 University Hospital of Lyon, 69500 Lyon, France; david.cheillan@chu-lyon.fr; 9 First Faculty of Medicine, Charles University, 128 08 Prague 2, Czech Republic; vera.frankova@lf1.cuni.cz; 10 Department of Genetics and Molecular Medicine—Newborn Screening, Landspitali University Hospital, 101 Reykjavik, Iceland; leifur.franzson@gmail.com; 11 University Hospital Centre Zagreb, 10000 Zagreb, Croatia; 12 Faculty of Medicine, University of Ljubljana, SI-1000 Ljubljana, Slovenia; urh.groselj@kclj.si; 13 Reference Laboratory for Neonatal Screening, Center for Health Protection, National Institute for Public Health and the Environment (RIVM), 3720 BA Bilthoven, The Netherlands; tom.van.den.kerkhof@rivm.nl; 14 Finnish National Newborn Screening Centre (Saske), Turku University Hospital, 20521 Turku, Finland; riikka.kurkijarvi@varha.fi; 15 Newborn Screening, Clinical Biochemistry and Clinical Pharmacy Lab Meyer Children’s Hospital IRCCS, 50139 Florence, Italy; 16 Department of Experimental and Clinical Biomedical Sciences, University of Florence, 50139 Florence, Italy; 17 Mother and Child Healthcare Institute of Serbia “Dr Vukan Cupic”, 11070 Belgrade, Serbia; 18 Norwegian National Unit for Newborn Screening, Division of Pediatric and Adolescent Medicine, Oslo University Hospital, 0424 Oslo, Norway; olvmol@ous-hf.no (O.M.);; 19 Laboratory Genetics, Neonatal Screening Center, 115580 Moscow, Russia; 20 National Institute for Mother and Child Health “Alessandrescu-Rusescu”, 050474 Bucharest, Romania; 21 Center for Preventive Paediatrics “Americos Argyriou”, 3022 Limassol, Cyprus; 22 Center for Pediatric and Adolescent Medicine, Department I, Division of Metabolic Diagnostics and Newborn Screening, Medical Faculty Heidelberg, Heidelberg University, 69120 Heidelberg, Germany; 23 Centre for Inherited Metabolic Diseases, Karolinska University Hospital, 171 76 Stockholm, Sweden; 24 Center for Molecular Medicine, Almaty 050020, Kazakhstan; 25 Pediatric Centre, Semmelweis University, 1083 Budapest, Hungary; 26 Institute for Children’s Diseases, Clinical Centre of Montenegro, 81000 Podgorica, Montenegro; majaraicevic89@gmail.com; 27 Department of Clinical Genetics, Genetics and Personalized Medicine Clinic, Tartu University Hospital, 50406 Tartu, Estonia; 28 Genetics and Personalized Medicine Clinic, Tartu University Hospital, 51014 Tartu, Estonia; 29 Department of Pediatrics, University Clinical Center Tuzla, 75000 Tuzla, Bosnia and Herzegovina; almatoromanovic88@gmail.com; 30 National Institute of Health Doutor Ricardo Jorge, Rua Alexandre Herculano, 321, 4000-055 Porto, Portugal; laura.vilarinho@insa.min-saude.pt; 31 Laboratorio de Metabolopatías y Cribado Neonatal, Hospital Regional Universitario de Málaga, 29011 Málaga, Spain; 32 Departamento de Biomedicina y Odontología, Universidad Europea de Andalucía, 29130 Málaga, Spain; 33 Austrian Newborn Screening, Department of Pediatrics and Adolescent Medicine, Medical University of Vienna, A-1090 Vienna, Austria; maximilian.zeyda@meduniwien.ac.at; 34 National Newborn Screening Laboratory, LabPlus, Health New Zealand Te Whatu Ora, Auckland 1023, New Zealand; dianne.webster@tewhatuora.govt.nz

**Keywords:** dried blood spot cards biobanking, informed consent, sample storage, European legislation, European general data protection regulation (GDPR), parental and children’s rights

## Abstract

Neonatal screening using Dried Blood Spot (DBS) cards is an important and successful public health facility in Europe, enabling early detection of congenital disorders for early treatment and prevention of overt disease. Biobanks of stored DBS cards offer significant potential for biomedical research. Biobanking and secondary use of DBS cards also raise ethical and legal issues, particularly concerning the rights of parents and children. This study provides a comprehensive overview of legislation and legal practices governing DBS biobanking across 29 European countries, based on a survey conducted in collaboration with the International Society for Neonatal Screening. Findings reveal a highly heterogeneous landscape: 15 countries have national legislation, five have regional guidelines, and nine lack formal regulations. Only four countries require explicit parental consent for DBS storage, with considerable variation in approval processes for research use. A harmonization of practices, with the European General Data Protection Regulation (GDPR) as a basis for future regulation, supplemented by clearer guidance on ‘public interest’ and robust safeguards for individual rights may lead to more transparent and consistent governance, which is essential to balance scientific progress with the protection of parental and children’s rights in DBS-based research across Europe.

## 1. Introduction

Neonatal screening is one of the cornerstones of public preventive health, ensuring that serious congenital disorders are identified within days of birth. This early detection allows for timely treatment, preventing severe disability or even death. The method is simple yet powerful: a few drops of blood from, usually collected from the heel of the baby on filter paper (a dried blood spot (DBS) card) shortly after birth, which enables laboratory analysis of a panel of disorders for which early treatment is available. The immediate intended use of the DBS cards is clinical, to ultimately diagnose and treat affected infants. Yet additionally, these DBS cards, containing DNA, RNA, proteins, and metabolites, hold great potential in secondary use for quality control, method development and biomedical research, both disease-oriented and population-wide, when stored in biobanks. For the purpose of this article, ‘biobank’ is the generic term for any storage of DBS cards, although it is recognized that ‘biobank’ can be defined differently depending on national legal and organizational regulations.

Denmark provides a striking example of the scientific value of such material. Newborn screening in Denmark is centralized at the Statens Serum Institute (SSI), where approximately 58,000 infants are screened each year for 25 congenital diseases [1]. Since 1981, all residual DBS cards have been stored systematically in the Danish National Biobank. Today, more than 2.5 million cards are preserved. In essence, this means that nearly every Danish citizen under the age of 45 has a sample archived [2]. This archive was initially intended primarily for quality assurance and retrospective diagnosis, but it soon became a foundation for research. Early studies demonstrated that DNA could be reliably extracted from filter paper and amplified, thereby opening the door to population-scale genetic research [3]. Indeed, research including genotyping, epigenetic profiling, next-generation sequencing, metabolomics, and RNA sequencing has meanwhile proven that these samples can be used in genetic studies [4,5,6,7]. Between 1981 and 2017, more than 50 projects have been approved, with over 180,000 DBS cards used for research [2]. The most notable of these, the iPSYCH project, genotyped 140,000 samples to explore the genetic and environmental basis of psychiatric disorders [8]. What makes this research particularly powerful is the ability to link biological data to nationwide registers through social security numbers. These registers encompass health records, demographics, education, income, and even life-course trajectories [9]. When combined with molecular data from the biobank, they enable studies on the interaction between genetics, environment, and social factors at a scale rarely possible elsewhere.

Yet such potential also comes with challenges. The use of residual DBS samples raises important ethical and legal questions and hence issues of consent, privacy, and the secondary use of material collected for clinical purposes are at the forefront of public debate [10]. Ultimately, there is the (rare) possibility that DBS cards are used for juridical purposes, demanding strict legal provisions. Although Danish law allows research use of DBS samples without explicit consent, provided that approval is obtained from a medical ethical review board, concerns have grown regarding transparency and data protection, particularly in light of the European General Data Protection Regulation (GDPR) [11]. The GDPR lists strict rules for organizations in the European Union (EU) that use personal data of individuals; it was established in 2016 and implemented in 2018.

These developments in Denmark trigger the research question of this study: How are similar issues handled in other European countries? It is well known that the practice of biobanking of DBS cards in Europe varies widely. A publication by the ISNS in 2021 indicated that 44 countries in Europe store DBS cards and 12 countries reported doing so with parental consent [12]. Another recent paper included a limited inventory of DBS card biobanks in South Eastern and Central European countries [13]. To further explore this, we evaluated in more detail how the biobanking and residual use of DBS samples is legally and ethically embedded in European countries, to identify good and best practice. In this study, we focus on two subjects: 1) the legal basis of biobanks and 2) the process of approval of an application for the secondary use of samples. We present an overview of the approaches in Europe and assess the potential for developing shared practice across Europe.

## 2. Materials and Methods

To investigate the international landscape of DBS storage and research use, a survey was conducted in collaboration with the International Society for Neonatal Screening (ISNS) [14].

### 2.1. Survey Instrument

The survey instrument was designed in MSForms (Microsoft, Redmond, WA) online version. The survey instrument consisted of 26 structured questions, designed to capture key aspects of both biobank infrastructure and governance (see Supplementary Materials).

Questions covered two main areas: storage practices and research use. Under storage, participants were asked whether DBS cards were kept after laboratory analysis for screening, whether and when consent is required (no specific distinction was made between opt-in and opt-out), when their biobank was established, how many samples were currently stored, and under what conditions (e.g., room temperature, −20 °C, etc.). They were also asked to report the age of their oldest stored cards.

The residual use of DBS cards was further examined through questions regarding the types of studies for which such use occurs. Additional questions addressed the ethical and legal frameworks governing the residual use of DBS samples, as well as the processes required for approval.

### 2.2. Study Population

The survey was distributed digitally on 11 December 2024 via an email link and was shared with a selected group of 64 participants in all European countries that were considered to be aware of the particulars of their NBS program across 51 countries. This group had previously been approached regularly to survey the current national and regional state of neonatal screening, and the summarized outcomes of their responses have been published previously [12]. Hence, this group was assumed to be able to also answer the questions on the survey of the current study. Nevertheless, respondents were encouraged to forward the survey within their professional networks to maximize the quality of responses. A reminder was sent to the non-respondents to motivate them to hand in their answers on 1 February 2025. After that, no additional reminders were pursued.

### 2.3. Processing and Analysis of Results

Results were collected in an online Excel sheet (MSExcel, Microsoft, Redmond, WA). Quantitative data (number of samples in biobank, oldest samples in biobank, storage time, storage temperature) were summarized and combined.

The outcomes of two research questions were analyzed qualitatively, namely:

- “What is the legal basis for residual use of DBS cards?” With the added instruction to be at liberty to answer in 3–10 lines and add links to prevailing rules and legislation if possible.
- “Please describe the process for approval to use DBS” again with the added instruction to feel at liberty to answer in 3–10 lines and also to elaborate on different approval grounds for different uses.

After the data compilation, author Sophie ter Braak and Christian Munch Hagen performed an initial narrative analysis and summary of the answers to the questions, especially to resolve small discrepancies in one instance in responses were received twice from the same respondent and one instance in which where answers were received for the same country from two different respondents. Then, this summary and narrative analysis were reviewed by the authors Peter Schielen and Mette Nyegaard to determine whether the summaries of the answers still reflected the original answers given by the respondents and that no meaning was lost. Summary results were mapped per country on a map of Europe.

When appropriate, information from respondents was used to find more elaborate information from open sources on the internet.

For presentation purposes, some truncated and abbreviated answers were adapted to make the text more readable (raw data is available upon request).

## 3. Results

After sending out the survey (December 2024) responses were received from respondents of 25 countries and regions and after one reminder (February 2025) four additional responses were received, totaling 29 countries/regions with responses. For Belgium, Germany, Hungary, Italy, Romania, and Spain, data from only some regions are available. NBS programs in these countries are organized regionally, and collecting data from all provinces of these countries was not feasible within the timelines of this study.

**Figure 1:**
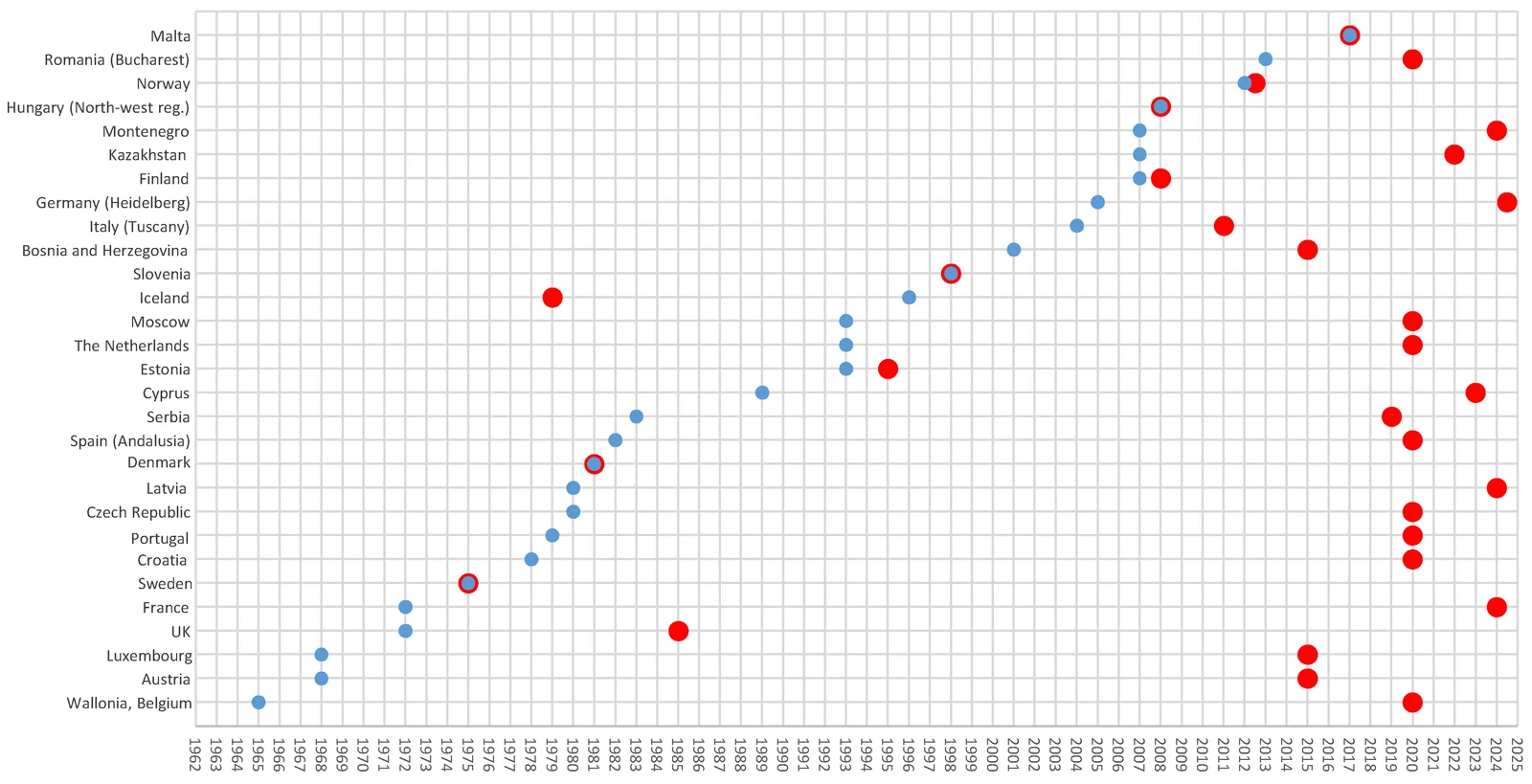
Overview of European countries with DBS card biobanks (sorted by year of establishment, blue symbols) and the storage year of the oldest cards in storage (red symbols; reference year: 2025).

Figure 1 shows an overview of European countries with biobanks and the age of the oldest samples in the respective biobanks. DBS cards can be stored in separate facilities or in the screening laboratory. These are all considered DBS card biobanks in the current study.

Screening organizations and laboratories in The United Kingdom, Iceland (56,000 samples from 1979–1991 stored in the biobank of Staten Serum Institut, Copenhagen, Denmark), and Sweden hold old samples, going back sometimes over 50 years, but in most countries, samples are stored for 1–5 or 10 years. In the Netherlands, a small selection of NBS cards of interesting (often: confirmed) cases are stored for 12 years (with parental consent, to be renewed by the child at 12 years of age). The Norwegian biobank for DBS cards was established in 2003 but legal changes in 2018 resulted in the destruction of all cards prior to 2012, and DBS cards from 2012 onwards have since been stored henceforth without a set end date.

*Relative and absolute size of biobanks*

**Figure 2:**
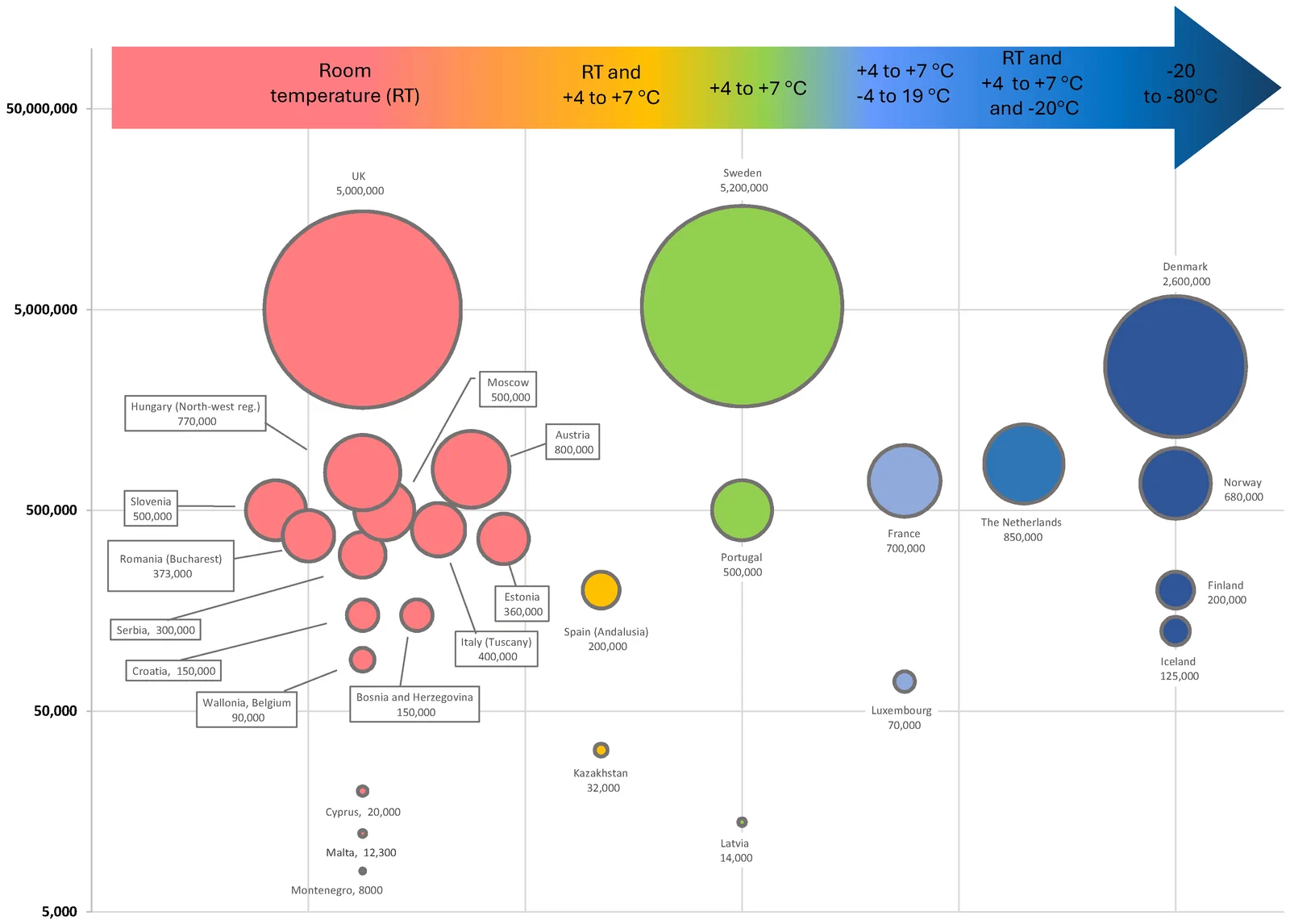
Number of DBS cards in biobanks in storage and storage conditions per country. The sizes of the biobank indicators (circles) are proportional to the total number of samples stored in each biobank. The circles are sorted along the horizontal axis from ambient to frozen. The color of the circles represents the storage conditions as presented by the matching color in the arrow. Note: Data from Germany (Heidelberg) and Czech Republic were not available.

Figure 2 gives an overview of the number of DBS cards stored in biobanks and storage conditions per country.

A majority of 16 out of 29 respondents from countries/regions indicate that NBS cards are stored at room temperature (RT), and another six at RT and/or 4 to 7 °C. Seven respondents indicate they store samples frozen (either at −4 to −19 C or −20 to −80 °C). Notably, many countries apply two (or even three; the Netherlands) storage conditions, e.g., a first period at RT or 4 to 9 °C, and a second period when samples are stored frozen, often at −20 to −80 °C.

Four of 29 respondents indicated that samples were stored under dry conditions (less than 30% humidity). Seventeen of 29 respondents indicated that retrieval data (name/address, demographics of the baby, sample code, etc.) were digitized for ease of access to samples. In the UK it is mandatory to physically separate demographic data from the uniquely coded stored cards.

The data from Figure 2, taken together, represent a lower limit of the total number of DBS cards stored in European countries and regions and provinces. Thus it can be calculated that a minimum of 20.6 million DBS cards are stored in Europe, of which 4.5 million (21.6%) are stored frozen.

The handling and issuing of samples from biobanks that contain millions of DBS cards will demand resources. To get an impression of the availability of such resources, we asked whether sufficient funds and personnel are provided to handle applications for samples. Fourteen of 29 respondents (48.3%) answered ‘No’ to that question. Nine of 29 (31%) indicated that they were properly funded and 6 indicated that the number of requests was low or that costs were covered by research projects, implying that 51.2% of respondents had no funding issues for managing the issuing of DBS cards.

*Legislative and regulatory aspects of biobanking*

**Figure 3:**
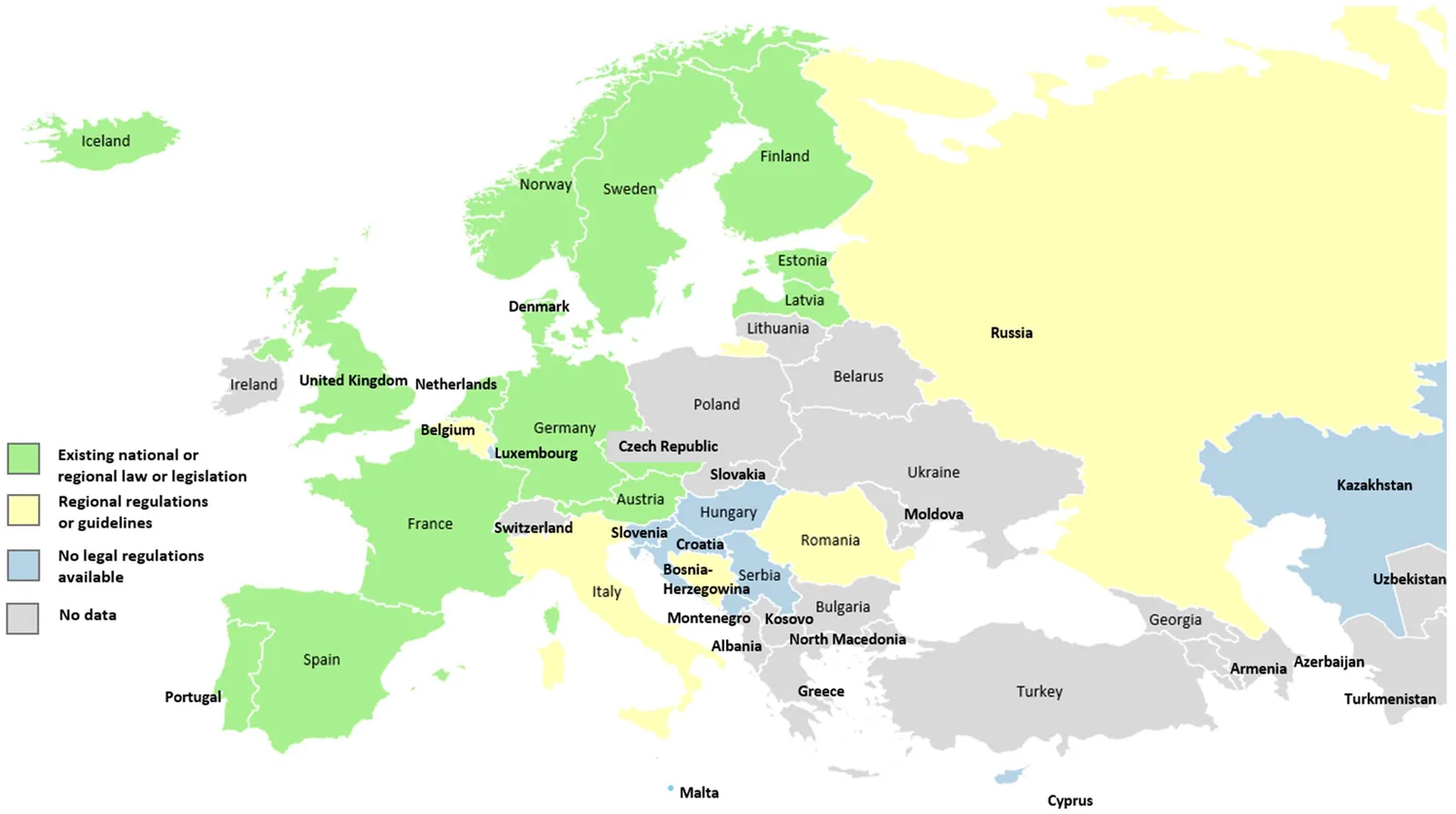
Legal basis for residual use of DBS cards in European countries. Green: countries with national legislation; yellow: countries with regulation at a regional level; blue: countries with no legal regulations present; and grey: no data received.

Table 1 and Figure 3 give an overview of various legislative and regulatory aspects of biobanking, where the color coding in both the table and Figure 3 is as follows: countries with national legislation: green; countries with regulations at a regional level: yellow; countries with no legal regulation present: blue; and no data received: grey. Of representatives of 19 of 51 countries, no reply was received.

Data from Table 1 illustrate that among the 29 countries included, 20 have a defined legal basis for residual DBS card use. Of these, 15 countries (in green in Table 1 and Figure 3) rely on nationally specified legislation, the Netherlands primarily also referring to the EU General Data Protection Regulation (GDPR). European law is considered here the highest order of law, as compared to national laws and regional regulation and guidelines. Five countries (in yellow in Table 1 and Figure 3), Belgium (more specifically; Wallonia), Bosnia and Herzegovina, Italy, Romania and Russia use guidelines of a lower order than national law and with only local applicability to guide biobanking and use of residual DBS cards. Legislation in Wallonia is again based on GDPR legislation. Sweden, France, Czech Republic, Spain, Germany, Estonia, Austria, Latvia, Iceland, Romania, the UK and Norway, follows national law specifically designed for biobanks and use of bodily tissues as their legal basis for residual use of DBS cards. Finland has national legislation that regulates the secondary use of samples and health data; however, the legislation does not contain any specific provisions regarding DBS cards. Respondents from Croatia, Cyprus, Hungary, Kazakhstan, Luxembourg, Malta, Montenegro, Serbia, and Slovenia indicate that they have no specific legal regulations for residual use.

*Consent for storage*

Three of 29 countries (Iceland, the Netherlands, and Spain) require explicit consent for a sample to be stored. In the Netherlands and Spain individuals retain the right to request removal of their DBS card (and associated data). In Norway and Sweden, consent is deemed to exist if the parents participate in the NBS program. In Norway, parents are recontacted when the child is two years old and the child at 16 years of age, informing them about the stored DBS card and indicating they can request destruction of the sample. In the Netherlands, for a small selection of samples stored for 12 years, consent needs to be renewed by the child when the child reaches 12 years of age. In Iceland, consent is irrevocable, but it is possible to request that samples are not used for research purposes. In Finland, the laboratory may store samples for internal quality control and method validation purposes. Under the Finnish Biobank Law, written consent is required for biobanking but samples stored at the laboratory are not considered a biobank under Finnish law. The Czech Republic constitutes a partial exception: DBS samples are mandatorily stored for five years, after which they are all destroyed.

*Formal procedures for re-use of stored DBS cards*

Regarding the approval processes for DBS card use, 24 countries have established formal procedures.

Four countries, Finland, Iceland, Latvia and Spain, require a high-order three-step approval for re-use for scientific purposes of DBS cards, namely individual or parental consent, ethics committee approval, and an additional form of authorization. Three countries (Czech Republic, Portugal and Italy) require at least parental consent and ethics committee approval. Five countries only require approval from an ethics committee, and three require only parental or legal guardian consent. Many different organizations can be involved in the authorization process, such as institutional approval in Latvia and Italy, application to the PKU Biobank in Sweden, Denmark and Spain, national committee approval in the UK, newborn screening reference group approval in Norway and detailed specified requests in Slovenia and the Netherlands. For medical purposes, retrospective analyses are generally accepted with either parental consent or a physician referral, or both (Sweden). It is of interest that most respondents indicate that stored DBS cards can be made the subject of legal procedure, but only by court order and applying very strict legal requirements.

Details of procedures in various countries give a more granular view of the many variations in approval procedures in Europe.

In Sweden, the approval process is dependent on the purpose. For instance, a physician’s referral, as well as patient or guardian oral consent, is needed for retrospective diagnosis for a patient while for research purposes it is mandatory to obtain approval from the Swedish Ethical Approval Authority, an approval which may be (but is not always) conditional on written informed consent. In Spain, for research purposes ethics approval, application to the PKU Biobank and parental consent is needed. For research with anonymized samples, however, such approval is not needed. In Estonia, the approval process is also dependent on the purpose. Secondary use for medical purposes or law enforcement requires parental permission, whereas use for laboratory quality improvements does not. Research additionally requires ethics committee approval. The same rules apply in Austria, in which ethical approval is required in the case of data reuse for research, however for quality assurance and cutoff studies no approval is needed. Residual use for research in Latvia requires approval from the ethics committee, the hospital, and the parents. Clinical tests require only oral parental permission.

## 4. Discussion

This study surveyed the legal basis for the residual use of DBS cards in Europe, and the procedures required for approval of such usage.

We invited 64 respondents from 51 European countries to fill in the survey. With representatives of 29 countries responding to the survey, we reached sufficient saturation to illustrate similarities and differences in European practice but we caution to overgeneralize; data of this study cannot be seen as being fully representative for all European countries and regions.

Data from Figure 1 and Figure 2 indicate that many European countries have biobanks and some have been founded decades ago. Roughly, biobanks mentioned in Figure 1 and Figure 2 together contain a minimum of 20.6 million samples, of which 4.5 million are stored frozen. From published literature [12,13] it can be derived that at least 18 other European countries have established biobanks, storing samples varying from less than 0.5 years to ‘indefinitely’, so the total number of stored samples in Europe may be many millions more.

This study shows that the vast majority of samples is stored at RT and only a minority of samples is stored at conditioned low humidity. It is well known, however, that most biomarkers measured in neonatal screening are vulnerable to high temperature and moist conditions [24]. The utility of massive storage of cards at RT may therefore be reconsidered, and replaced with a policy to store less DBS cards and matched controls but under superior storage conditions (dry and at −20 to −80 °C).

The findings of this study provide insight into how European countries navigate the balance between scientific opportunities, individual rights, and public trust. They also illustrate a heterogeneous regulatory landscape across Europe, highlighting the variation in legal frameworks and approval requirements for DBS card utilization, with possible gaps in medical ethical standards for biobanking and re-use of DBS cards.

The diversity in approval requirements suggests different ethical priorities. Countries emphasizing parental consent appear to place autonomy and family control at the center of decision-making. Others that allow access through ethics committee approval or institutional authorization may prioritize collective benefit and the facilitation of research. Systems requiring multiple levels of authorization, such as those in Finland, Iceland, Latvia and Spain, but also Norway, Sweden, Denmark and the Netherlands, reflect attempts to combine safeguards, but they also introduce procedural complexity that may hinder research efficiency.

The heterogeneity of governance has significant practical implications. Inconsistent standards make multinational research collaborations unmanageable, particularly for large-scale studies of rare diseases where cross-border sample sharing is necessary. Researchers may face additional administrative burdens, delays, or exclusions depending on the country of origin of the DBS samples. Conversely, permissive frameworks that minimize parental involvement risk generating mistrust, as illustrated by Denmark’s suspension of new DBS research projects after public concern, despite following the legal rules [10,25].

The findings underscore the pressing need for greater clarity and harmonization. Fragmentation not only complicates the work of researchers but also threatens the trust in neonatal screening programs themselves if parents perceive a lack of transparency regarding how residual samples are used. Effective governance must therefore strike a careful balance between safeguarding individual rights and enabling collective scientific progress. The legal practice of storing DBS cards in biobanks inevitably implies the management of personal data. Future regulation at the European level could build upon the GDPR. Since 25 May 2018, all member states should follow the European Data Protection Regulation with the goal to harmonize data privacy laws across Europe [11]. Both Belgium and the Netherlands base the residual use of individual data on the GDPR (Table 1).

Under the GDPR processing of personal data (including genetic and biometric data and health data) is generally prohibited, but there are several exceptions [26]. One of these exceptions is that data may be used for preventive or occupational medicine; for public health reasons, such as serious cross-border threats to health or ensuring safety of medicinal devices; and for archiving, scientific, historical, or statistical research purposes in the public interest, provided it protects fundamental rights also included in the GDPR [27].

In the context of the results of this study, depending on what country is considered, several of these exceptions may apply. The first pertains to situations in which explicit consent is obtained from the data subject or, where relevant, their legal guardian. A second exception arises where processing is justified on grounds of substantial public interest or public health. Lastly, data derived from DBS cards may be processed for scientific or statistical research purposes in the public interest, provided that such processing is conducted in accordance with Article 89 of the GDPR. This article requires that the use of identifiable data is limited to what is strictly necessary and demands the implementation of appropriate safeguards. Such safeguards include the application of pseudonymization, whereby identifiers are replaced with codes, or, where feasible, full anonymization. When anonymized data makes the research impossible, an exception can be made [27]. Realizing that many of the countries surveyed in this study are not European Union member states, we incline here that the provisions of the GDPR could help to build national or even regional frameworks for biobanking of DBS cards, respecting parents and child rights and still facilitating collaborative research to improve screening programs for all European countries. Building future regulation on the GDPR also presents certain challenges. Countries that rely mainly on GDPR face greater interpretative challenges, as GDPR was not designed with biobanking specifically in mind [11]. Its ideas about sensitive personal data and research exemptions are open to differing interpretations. Future initiatives should therefore place particular emphasis on clarifying the concept of ‘public interest,’ since this determines whether personal data may be used without consent. Rather than creating new laws, efforts should focus on providing clearer and more detailed guidance within the framework of the GDPR.

One facility that may become important in the near future to standardize the personal data underlying biobanking in European countries is the European Health Data Space (EHDS) [28]. Combining the legislation of the GDPR, and the European Data Governance Act, Data Act and Network and Information Systems Directive, the EHDS will facilitate the access of data and materials in European biobanks, guaranteeing the rights of European citizens, still enabling scientific research in a responsible way.

One other organization that may hold important information, expertise and support to better organize biobanks of DBS cards in Europe is the Biobanking and BioMolecular resources Research Infrastructure—European Research Infrastructure Consortium (BBMRI-ERIC; https://www.bbmri-eric.eu accessed on 5 April 2026) [29,30]. BBMRI-ERIC was established in 2013 under EU legislation and enables the development of innovative technology and processes as a cross-domain network that facilitates responsible access to high quality samples, data and biomolecular resources. Finally, the ISNS in 2025 has issued updated guidelines for neonatal bloodspot screening, including guidelines for biobanking [31].

The situation in Denmark regarding the storage and residual use of DBS cards was the starting point of this research. Access to biological samples from the Danish National Biobank (DNB) follows a structured approval process: researchers must first obtain permission from both a Danish Research Ethics Committee and their research institution, after which the DNB Coordinating Centre grants final authorization based on a project description and a detailed specification of the requested samples. This framework aligns with the requirements of the GDPR, which permits the processing of sensitive data when research is considered to be in the public interest. Consequently, the iPSYCH project, assuming to be in the public interest, is regarded as GDPR-compliant, even though it gave rise to public mistrust. The extent to which further safeguards were implemented to reduce the risk of re-identification remains difficult to conclude.

Limitations of this study are that data were gathered from twenty-nine countries, so not including all European nations, and possibly missing intra-country variation, particularly in regionally governed systems. In addition, sometimes participants responded for a region, province or even city within a country, so the data can be used only to a limited extent as an approximation for the entire country. Second, the data were self-reported by national representatives, which introduces the risk of bias, incomplete responses, or inconsistencies. Third, legal frameworks are evolving; for example, draft laws in Slovenia and Serbia are likely to reshape their governance landscapes.

## 5. Conclusions

In conclusion, this study reveals that while many European countries have taken steps to establish governance mechanisms for DBS use, the overall landscape remains fragmented. Without a clearer and more consistent framework, Europe may struggle to fully realize the scientific potential of DBS collections. The way forward lies in developing more clearly stated rules, based on the GDPR and using the groundwork already developed by BBMRI-ERIC and EHDS. Such a framework should integrate strong safeguards for individual rights while facilitating collaborative biomedical research. Given that screening disorders are rare disorders, European collaboration is necessary and ultimately, DBS-based research can only proceed and succeed if governance across Europe is transparent, trustworthy, and fair.

## Figures and Tables

**Table 1:** Summarized answers of 29 European countries, representing national or regional responses to the survey questions: “What is the legal basis for residual use of dried blood spot cards?” and “What is the process for approval to use dried blood spot samples?” Green represents the presence of national law, yellow the presence of regional regulations, and blue the absence of any regulation. PKU = phenylketonuria; PKU Biobank stores the dried blood spot DBS cards. CMV = cytomegalovirus.

Country	Legal Basis for Residual Use of Dried Blood Spot (DBS) Cards?	Consent and Removal of DBS Cards		Process for Approval to Use DBS Cards		
		Consent for Storage Needed	Parental/Guardian Request for Removal or Return Enabled	Research	Quality Assurance/Method Development	Juridical Purpose
**Existing national or regional law or legislation**						
Austria	The “Research Organization Law”.	No	Yes	Requires local Ethics Committee approval.	No approval.	No data.
Czech Republic	Stored for 5 years (Decree No. 98/2012 Coll.). Anonymized residual use allowed (Act No. 372/2011 Coll.). Genetic research requires guardian consent (Act No. 373/2011 Coll.).	No	Not within the five years of mandatory storage	Ethics Committee approval required for all research using DBS samples, additionally parental (legal guardian) consent required when samples are identifiable (e.g., pseudonymized).	No consent required.	Requires court order.
Denmark	From 1993 regulated as part of The Public Register Act. Updated in 2002 with the “An account of Biobanks” which included implementation of the EU-directive 95/46/E [2,15].	No	Yes	Application to the Danish National Biobank and approval from the Danish Research Ethical Committee, followed by application to the ‘PKU biobank [16].	No consent required.	Requires approval from a judge and only to identify victims, biobank cannot be used to identify criminals.
Estonia	Estonian legislation.	No	Yes	Requires ethics committee approval.	No consent required.	Requires parent/guardian permission.
France	French law requires NBS samples to be stored for at least one year for result confirmation, further analysis, or research with parental consent [17].	No	Yes	Reuse only with parental consent on request.		
Finland	Finnish Law on the Medical Use of Human Organs, Tissues, and Cells (paragraph 20 -Change in the Intended Use of the Samples) regulates the secondary use of samples collected for diagnostic and therapeutic purposes (applicable to DBS cards; no specific legislation for DBS cards only). Finnish Law on the Secondary Use of Social and Health Data.	No	Yes	No specific process for DBS cards, general national and local regulations applicable. Research use generally requires consent. Institutional approval required for research. Approval by the ethics committee needed depending on the research type.	No consent required.	No data.
Germany	Kinder-Richtlinie [18].	No	Yes	Residual use only allowed with prior parental consent.		
Iceland	Law on Biobanks and Health Information 2000/110. Law on Health Research 2014/44. Law on the Rights of Patients 1997/74.	Yes	Not destroyed, but parents can request (through the homepage of the Directorate of Health) that cards are not being used for further research	The law allows secondary use of samples for patient benefit, assay development, training, quality control. Depending on type of research demands (1) approval by hospital Ethical Review Board, (2) Data protection agency, (3) application to the biobank and (4) application to Director of the hospital.	Non-clinical use must be anonymized.	No data.
Latvia	Cabinet of Ministers Regulation No. 611 “Procedure for providing obstetric support” [19]. Cabinet of Ministers Regulation No. 9 “Regulations of the Central Medical Ethics Committee” [20]. Cabinet of Ministers Regulation No. 698 “Regulations of the Genome Research Council” [21].	No	Yes	Research needs Ethics Committee and hospital approvals plus parental consent	No data.	No data.
Netherlands	(1) GDPR(/AVG); article 9, paragraph 2, and article 89, (2) Dutch law; Population Screening Act and Medical Treatment Agreement Act.	Yes (but not needed for quality assurance)	Yes	Parental consent needed. Requests via established procedure through Research committee, following general terms and conditions.	No consent required.	With court order and very strict legal requirements. Has never occurred in the history of the Dutch biobank.
Norway	Regulations on Genetic Mass Screening of Newborns, paragraph 3 Storage and use of blood samples. The blood samples from the mass screening shall be stored in a diagnostic biobank, cf. the Biobank Act § 2, first paragraph. Biobank law for healthcare; paragraph 9a states reuse for healthcare, quality assurance, method development or health research only. Research requests should be in accordance with the Health Research Act.	No	Yes	Requires ethics approval, participant consent, and NBS reference group approval.	Stored samples may be used for healthcare, quality control, and method development.	Case law may be applicable.
Portugal	DBS cards are kept for 5 years for lab quality control; other uses need parental consent (no specific legislation mentioned).	No	Yes	In addition to consent, research use requires ethics committee approval.	No data.	No data.
Spain	DBS cards can be stored and used for at least five years for neonatal screening purposes without written consent, based on Spanish laws (Law 41/2002, Law 14/2007, RD 1716/2011) and data-protection regulations. Use for other purposes, like research, requires explicit written consent.	Yes	Yes (individuals/legal guardians) can request removal of sample and associated data)	Application to PKU Biobank and ethics approval and consent (unless anonymized).	No consent required.	Law enforcement demands judicial authorization, strict purpose limitation, and oversight by legal counsel and the data-protection officer.
Sweden	The Swedish biobank law: “Biobankslag (2023:38)” [22,23].	No	Yes (removal only, there is no legal framework for return of the sample to the donor/guardian)	Approval from Swedish Ethical Review Authority and application to the ‘PKU biobank’ (Swedish DBS-card biobank).	No consent required.	Judicial purposes are not a legal use of DBS cards according to the biobank law, except to identify deceased individuals (which has a specific and separate process).
United Kingdom	Compliance with the Human Tissue Act in the UK.	No	Yes	Research needs national committee approval.	Method development needs national committee approval.	Law enforcement follows laboratory legal guidance.
**Regional regulations or guidelines**						
Belgium (Wallonia region)	DBS cards are stored for 5 years, used only for defined follow-up purposes or compatible new studies if aligned with the GDPR.	No	Yes	Residual use requires submission to the competent authority with detailed justification, objectives, and methods.		
Bosnia and Herzegovina	The University Clinical Center Tuzla has a written procedure for DBS storage and residual use.	No	No	No formal guidelines.	No data.	No formal guidelines.
Italy (Tuscany Region)	In Tuscany, DBS are stored for 10 years for NBS or legal use; secondary use needs family consent.	No	Yes	Research requires consent and approval from institution review board.	No data.	Law enforcement needs court authorization.
Romania (Regional screening center Bucharest)	“National Guide for screening, diagnostic and treatment of phenylketonuria” issued in 2016.	No	No	No official process.		
Russia (Moscow)	Local guidelines define storage/reuse.	No	No	Used for missed cases or new disease screening.		
**No legal regulations available**						
Croatia	No legal regulation.	No	Yes	Secondary research requires ethics committee approval; CMV testing only needs medical staff approval.	No data.	No data.
Cyprus	No legal regulation.	No	Yes	Secondary research requires approval from the National Bioethics Committee.	No formal approval necessary for any quality assurance practices on the stored samples. Method development is not within the scope of card storage.	Release sample for juridical purposes subject to court’s decision.
Hungary (Northwest Region)	No legal regulation.	No	Yes, by written request	Written request from authorized medical staff required for CMV analysis.		
Kazakhstan	No legal regulation.	No	No		Interlaboratory comparisons and confirmatory testing follow Order of the Minister of Health of the Republic of Kazakhstan No. QR DSM-91 dated 25 August 2021.	Residual use occurs only upon official court request.
Luxembourg	No legal regulation.	No	Yes	No official process		
Malta	No legal regulation.	No	No	No official process		
Montenegro	No legal regulations.	No	No	In the Institute for Children’s diseases (Clinical center of Montenegro) cards may be used for other metabolic tests without approval	No data	Laboratory head handles law-enforcement requests.
Serbia	Draft regulation in progress.	No	No	No official process		
Slovenia	Draft regulation in progress.	No	Yes	Secondary use only with request and ethics approval		

## Data Availability

An Excel file with all raw data is available upon request to peter.schielen@isns-neoscreening.org.

## Supplementary Materials

The following supporting information can be downloaded at: https://www.mdpi.com/article/10.3390/ijns12030064/s1, Survey: 20260505_ISNS_survey_biobanking_2024–2025.
