# Supplementary material for: Features and Legal Practice of Dried Blood Spot Card Biobanking in Europe: Balancing Research Potential with Parental and Children’s Rights
Source: Int J Neonatal Screen. 2026 Aug 5;12(3):64. doi: 10.3390/ijns12030064 (PMC13510356; doi:10.3390/ijns12030064)
Supplement: Supplementary file 1 [file IJNS-12-00064-s001.zip › IJNS-4333211-supplementary.pdf]

ISNS. NBS screening card storage: facilities, management, consent, legal

L.S.

The ISNS invites you to participate in a survey on storage of dried blood spot samples (NBS screening cards) and their use and issuing of samples. We will ask questions in groups on the following subjects: 1)-general, 2)-consent for **storage of** DBS screening cards, 3) length of storage, 4)-specifications of the storage facility, 5)-secondary use of stored dried blood spots, 6)-consent for **secondary use** of DBS screening cards and 7)-Legal basis for storage and permission for secondary use.

We are aware of the fact that there are many forms of and definitions for storage of NBS samples, from storage in the laboratory and storage in a general off-site facility to storage in a regulated, high-tech facility or a combination. Sometimes, these are called 'biobanks' and biobanks sometimes laws define what biobanks are. Please use your own definition of 'Storage of NBS screening cards' here and use the free text boxes to give a more detailed explanation if needed. The text boxes will allow for texts up to 5-10 sentences, just keep typing.
If you do not have an answer to a question, tick the 'Oher' box and type 'No answer'.Please answer all questions from the perspective of your regional or national screening program.

Through this survey, ISNS gains a comprehensive overview on storage of NBS (which it does not currently have), to share with our membership. The survey will take 15-20 minutes. Your help in this matter is greatly appreciated.

Your personal data

1. Full name

2. Affiliation

3. Email address

4. I am filling in this survey for (enter country or region/province)

General

5. Are DBS screening cards stored for any period of time after the screening analysis? *

- ◯ No
- ◯ Yes

DBS screening cards are not stored

Please answer this question if DBS screening cards are not stored

6. How many days after analysis are DBS screening cards destroyed? *

- ◯ About 14 days after
- ◯ 14- 30 days after
- ◯ 30-90 days after
- ◯ Other

Consent for storage of NBS screening cards

7. Do parents/sample donor/guardian need to give consent for storage (at 'other': add free text if answer needs more elaboration) *

- [ ] No
- [ ] Yes, and parents/guardians need to opt OUT by checking a box (or have box checked) on the DBS screening card
- [ ] Yes, and parents/guardians need to opt OUT by signature, writing initials, or through digital portal/request
- [ ] Yes, and parents/guardians need to opt IN by checking a box (or have box checked) on the DBS screening card
- [ ] Yes, and parents/guardians need to opt IN by signature, writing initials, or through digital portal/request
- [ ] Other

8. Does the consent need to be updated , e.g. at a certain age (at 'other' give more details if necessary, e.g. through written letter, client portal, update at 2 and 16 years of age, etc.) *

- [ ] No
- [ ] Yes
- [ ] Other

9. Can the parents/sample donor/guardian ask for the NBS screening card to be destroyed (At ' Other', give more details if necessaray; e.g. through portal, written request/other) *

- [ ] No
- [ ] Yes
- [ ] Other

10. Can the parents/sample donor/guardian ask for the NBS screening card to be returned to them (At ' Other', give more details if necessary; e.g. through portal, written request/other) *

- [ ] No
- [ ] Yes
- [ ] Other

How long are NBS screening cards stored

We also ask when storage of NBS screening cards started, and what the oldest

11. Please fill in the specifications of the storage (at 'other' add details, e.g. 2 years, but samples of cases 12 years). Multiple or all answers possible.

- [ ] 1-3 years
- [ ] 4-6 years
- [ ] 7-20 years
- [ ] 20 years-indefinite
- [ ] Other

12. In what year was the storage of NBS screening cards initiated (e.g. 1975, 1990, etc)

13. How old are the oldest NBS screening cards in your storage facility (e.g. 6 years, 14 years etc..)

14. What is the total number of NBS screening cards in your storage facility (e.g. 50.000, 1.2 milion etc.)

Specifications of the NBS screening cards storage facility

15. Which of the following statements concerning the specifications of your NBS screening card storage facility apply. Multiple or all answers possible.

- [ ] room temperature
- [ ] 4-7 degrees Celsius
- [ ] -4 to -19 degrees Celsius
- [ ] -20 to -80 degrees Celsius
- [ ] controlled humidity to 30% or less
- [ ] automated storage rooms, e.g. with robots for easy retrieval of samples
- [ ] sample information (name/address, demographics of the baby, sample code, etc.) digitized for easy retrieval
- [ ] Other

16. Concerning the physical accessibility and safety measures of your NBS screening card storage facility, which items apply (Multiple or all answers possible. At 'other' add any other items that apply)

- [ ] Only personnel with security clearance has access
- [ ] Access protected using personal security codes or security pass/other form of digital authentication
- [ ] Access is recorded
- [ ] Access only in working hours
- [ ] Other

Use of dried blood spot samples

17. For what use can DBS screening cards be issued (at 'other' please add free text if answers need more elaboration). Multiple or all answers possible. *

- [ ] Quality assurance (e.g. confirmation of original laboratory result)
- [ ] Determination of cut-of-values for the routine neonatal screening program
- [ ] Confirmatory diagnostic testing
- [ ] Interlaboratory comparison programs
- [ ] Development of new screening methods
- [ ] General not-NBS related research questions
- [ ] Research and development with commercial partners
- [ ] To investigate other conditions in the child/baby with the agreement of the parents eg CMV testing
- [ ] Other

18. Are there any guidelines that determine what secondary research is approved (for example, favoring research where the condition is early in life, disapproving late-onset conditions, etc.- (at 'other' please add free text if answer needs more elaboration). *

- [ ] Yes
- [ ] No
- [ ] Other

19. Are there any guidelines on how much sample (e.g. one full blood spot) should be left on the card, before it can no longer be included for any secondary research (at 'other' please add free text if answer needs more elaboration). *

- [ ] Yes
- [ ] No
- [ ] Other

20. In what circumstances and with what protections can DBS cards be accessed by Law enforcement agencies (at 'other' please add free text if answers need more elaboration) *

- [ ] They cannot accessed by law enforcement agencies and this is protected by national law
- [ ] They can be accessed by law enforcement agencies to establish the identity of deceased remains unrelated to specified criminal investigations
- [ ] They can be accessed by law enforcement agencies when pursuing a criminal investigation upon direct request from the law enforcement agency
- [ ] They can be accessed by law enforcement agencies when pursuing a criminal investigation only with a ruling from a Court to require access
- [ ] They can be accessed in pursuance of civil litigation such as paternity investigations
- [ ] Other

Consent for secondary use of DBS screening cards

21. Is consent for secondary use of DBS screening cards required (at 'other': add free text if answer needs more elaboration) *

- [ ] No
- [ ] Yes, and parents/guardians need to opt OUT by checking a box (or have box checked) on the DBS screening card
- [ ] Yes, and parents/guardians need to opt OUT by signature, writing initials, or through digital portal/request
- [ ] Yes, and parents/guardians need to opt IN by checking a box (or have box checked) on the DBS screening card
- [ ] Yes, and parents/guardians need to opt IN by signature, writing initials, or through digital portal/request
- [ ] Other

22. In Denmark parents/sample donor/guardian can ask for the DBS screening card to be exempt from being included in research. This data is then kept in a national register. Each time a project is asking for punches, this register is checked and if parents/sample donor/guardians are in that register, no material will be issued for that research. *

- [ ] Yes, in my country we have a similar system
- [ ] No, such a register does not exist in my country
- [ ] Other

23. What of the following statements on issuing of DBS screening cards applies (multiple answers possible)

- [ ] One to a few punches are issued
- [ ] One blood spot is issued
- [ ] A complete card is issued
- [ ] demographic data be included with the sample to the applicant
- [ ] Material needs to be discarded after use
- [ ] Material needs to be returned after use
- [ ] Material cannot leave the laboratory - analysis for research and development will be carried out in the laboratory
- [ ] Material cannot leave the country
- [ ] Material cannot leave the EU
- [ ] Results of analysis are reported back to the laboratory
- [ ] Parents/donors/guardians are informed of the use of their sample for secondary or tertiairy use

24. Are sufficient resources (financial and personnel) provided to handle applications for samples *

- [ ] No
- [ ] Yes
- [ ] Other

Legal basis for storage and due process for approval to issue DBS screening cards

25. What is the **legal basis** for storage and secondary use of residual DBS screening cards? Please answer in 3-10 lines and add links to prevailing rules and legislation if possible. *

26. Please describe the **process** for approval to use DBS screening cards (for any or all items in question 17 (secondary use of NBS screening cards) and question 20 (use of screening cards by law inforcement agencies). Please answer in 3-10 lines and add different conditions for different uses. Please mention independent regional, central or laboratory approval boards, formal written application process, staff approving of use, etc. *

Closing question

27. Is there anything else relevant to biobanks of DBS that you would like us to know

This content is neither created nor endorsed by Microsoft. The data you submit will be sent to the form owner.

Microsoft Forms
